# Unpredictable Feeding Impairs Glucose Tolerance in Growing Lambs

**DOI:** 10.1371/journal.pone.0061040

**Published:** 2013-04-16

**Authors:** Anne L. Jaquiery, Mark H. Oliver, Nina Landon-Lane, Samuel J. Matthews, Jane E. Harding, Frank H. Bloomfield

**Affiliations:** 1 Liggins Institute, University of Auckland, Auckland, New Zealand; 2 Waikato Clinical School, University of Auckland, Hamilton, New Zealand; 3 Department of Paediatrics: Child and Youth Health, University of Auckland, Auckland, New Zealand; 4 Gravida: National Centre for Growth and Development, Liggins Institute, Auckland, New Zealand; Broad Institute of Harvard and MIT, United States of America

## Abstract

Irregular eating is associated with insulin resistance and metabolic disease in adults but may affect young, growing children differently. We investigated the metabolic effects of unpredictable feeding in female juvenile lambs randomly assigned to receive, for six weeks, maintenance feed given twice daily in equal portions (Control Group, C; *n* = 24) or the same weekly feed amount in aliquots of variable size at unpredictable times (Unpredictable Group, U; *n* = 21). Intravenous glucose tolerance tests (IVGTT), insulin tolerance tests (ITT), and measurement of diurnal plasma cortisol concentrations were performed pre and post the dietary intervention. Groups were compared using t test and RM ANOVA. Weight gain was similar in both groups (C 18±2%; U 16±2% of initial body weight). Glucose area under the curve (AUC) was unchanged in C (AUC pre 818±34, post 801±33 mmol.min.l^−1^), but increased by 20% in U (pre 830±25, post 1010±19 mmol.min.l^−1^; p<0.0001), with an inadequate insulin response to glucose load (log(AUC insulin first 40 minutes) post intervention C 1.49±0.04 vs U 1.36±0.04 ng.min.ml^−1^; p = 0.03). Insulin tolerance and diurnal variation of plasma cortisol concentrations were not different between groups. Unpredictable feeding impairs insulin response to glucose in growing lambs despite high quality food and normal weight gain. Irregular eating warrants investigation as a potentially remediable risk factor for disordered glucose metabolism.

## Introduction

Altering meal frequency, particularly by skipping meals and snacking when not hungry, affects the physiological response to a meal in ways that promote weight gain even if caloric intake is not substantially increased [1], [2]. However, there is less information about the physiological effects of an irregular food supply, where meal frequency and the amount of food eaten vary unpredictably from day to day. A population-based cross-sectional study in adults found that those who reported eating regular meals had a lower incidence of metabolic syndrome, although such studies cannot determine if these relationships are causal [3]. A small crossover study in nine lean women who ate regularly, then irregularly, for two week periods demonstrated insulin resistance and higher fasting lipid profiles during the period of irregular eating [4]. Observational studies in children suggest that skipping meals, particularly breakfast, is a risk factor for adolescent obesity [5], [6] and leads to higher baseline plasma insulin and low density lipoprotein concentrations, [7] thereby potentially contributing to an increased risk of cardiovascular disease over time. While many of these studies investigated eating pattern, satiety and nutritional adequacy of the diet, few studied metabolic consequences other than obesity.

The importance of circadian rhythms in the development of metabolic syndrome increasingly has been explored, including the role of the hypothalamic-pituitary-adrenal (HPA) axis and normal diurnal variation in plasma cortisol secretion [8], [9]. For example, persistently high plasma cortisol concentrations have been associated with increased insulin resistance in adults [10]. Normal cortisol secretion varies with sleep- wake cycles, but is also affected by the timing of food intake, in both humans [11] and animals [12], [13]. Snacking in humans increased salivary cortisol concentrations even if the food was not taken in response to hunger [14]. Delaying feeds beyond the usual time in animals fed once daily resulted in increased plasma cortisol concentrations, suggesting a stress response [15]. Restricting feeding to a certain time of day in rodents resulted in increased anticipatory behavior, including an increase in corticosterone secretion, prior to the feed [16], an effect which appeared to be mediated by entrainable circadian oscillators in peripheral tissues, rather than affecting the suprachiasmatic nuclei (SCN) in the hypothalamus [17]. However, when food availability returned to normal, peripheral oscillators were then reset. Less clear are the effects of unpredictable or variable food intake on ciracadian rhythms and HPA axis function.

Young children are dependent on others to provide their food, making them susceptible to eating patterns that are neither predictable nor matched to their hunger signals. Nutrition surveys have shown that even in relatively affluent countries, up to 50% of households in some population groups report running out of food ‘sometimes’ or ‘often’, so that a consistent food supply may not be provided to the children living within them [18], [19]. Furthermore, it has been demonstrated that food insecurity is associated with maternal-infant feeding styles more likely to result in obesity [20]. The metabolic effects of irregular eating in otherwise healthy, rapidly growing children may be different from those in adults, and not necessarily associated with adverse metabolic outcomes. However, there are few data from studies in children and dietary experiments of this kind are not ethical in young children.

To investigate the metabolic effects of an irregular food supply in early life we therefore performed a study using healthy growing prepubertal lambs. Although sheep are ruminants, they adjust their circadian rhythms to the time of feeding when housed indoors; and the ontogeny of ovine glucose metabolism [21], the response to insulin-induced hypoglycemia by mobilisation of free fatty acids, and ovine HPA axis function [22] have all been been well documented. We hypothesised that food given at unpredictable times in variable aliquots for a six week period would affect glucose tolerance, insulin sensitivity, and diurnal cortisol rhythmicity, independent of caloric intake.

## Materials and Methods

Ethical approval for all aspects of the study was obtained from the University of Auckland Animal Ethics Committee (AEC number R767). Female offspring of Romney ewes were weaned at 12 weeks. From 16 weeks of age, lambs were housed indoors in two large group pens within a photoperiod controlled feedlot (indoor lights on between 7 am and 7 pm, with windows on the external walls also allowing natural light; 13–18 animals to any one pen during the experiment) and acclimatized over a week to a feeding regimen using HNF®Fiber feed (Fiber Fresh Feeds Ltd, Reporoa, NZ), a lucerne based silage with dry matter metabolisable energy of 11 MJ/kg and crude protein content of 22%, in amounts calculated to allow weight gain of 100–150 g/day with equal portions given in the morning (8–9 am, 1–2 hours after the lights went on) and late afternoon (4–5 pm). After acclimatisation, lambs were randomly assigned to one of two groups by sequential removal of obscured tag numbers from an opaque container: Control Group (C, n = 24), receiving feeds twice daily as previously; or Unpredictable Group (U, n = 21), receiving the same total weekly amount of feed, but given 0–3 times a day, in unequal portion sizes according to a pre-prepared random fortnightly schedule to ensure equal amounts were given to each group by the end of each week. Feed portions were given to the U group at any of 4 times: 8–9 am, 12–1 pm, 4–5 pm and 8–9 pm. The two group pens were in the same building but separated by a 1.5 m raceway. Actual feed intake in each group was monitored by weighing any leftover food each morning before the first feed. Animals were weighed twice weekly. At the end of the six week intervention period, animals were moved to individual pens for the tests detailed below and all animals were fed twice daily as for the C group.

Before and at the end of the six week intervention period, all lambs had jugular venous catheters inserted using local anesthetic [23]. After a recovery period of 12 hours, blood samples were taken every 2 hours for 24 hours for measurement of the diurnal pattern of cortisol concentrations. After a further recovery day, an intravenous glucose tolerance test (IVGTT) and an insulin tolerance test (ITT) were performed on consecutive days [24]. Animals were fasted overnight with free access to water. Tests were performed in the morning, and the morning feed given on completion of the test, with a second feed in the late afternoon. For the IVGTT, baseline samples were taken, then 0.5 g.kg^−1^ 50% dextrose was given intravenously over 30 seconds. Blood samples were collected at 2, 5, 10, 15, 20, 30, 40, 50, 60, 90, 120, 150 and 180 minutes post injection. For the ITT, baseline blood samples were taken, then insulin 0.15 IU.kg^−1^ was given as an intravenous bolus, and blood samples collected at 2.5, 5, 10, 15, 20, 30, 40, 50, 60, 80, 100 and 120 minutes post injection.

Blood samples were collected on ice, centrifuged for 10 minutes at 4°C at 3000 rpm, the plasma separated and frozen at −20°C until assay. Plasma cortisol concentrations were measured using mass spectrometry; [25] inter and intra-assay coefficients of variation (CVs) were 5.7% and 3.5% respectively. Glucose and free fatty acid (FFA) concentrations were measured using enzymatic colorimetric assay (Hitachi 902 Automatic Analyser; inter- and intra-assay CVs: glucose 2.5% and 1.4% respectively; FFA 3.4% and 3.2%). Plasma ovine insulin concentration was measured by radioimmunoassay using ovine insulin as the standard (Sigma Chemical, St Louis, MO, USA; inter- and intra-assay CVs 9.4% and 7.9% respectively) [26], [27]. Areas under the curve (AUC) were calculated for both glucose and insulin concentrations using a triangulation method.

Data were analysed using Statview, version 5, SAS Institute Inc., Cary, NC, USA). Where data were not normally distributed, log transformation was performed prior to analysis. Groups were compared using unpaired and paired t tests where appropriate, and changes over time were analysed using repeated measures (RM) ANOVA. Cortisol variability was analysed by calculating the standard deviation (SD) of cortisol concentrations over the 24 hour period for each animal separately; these data then were compared between the two groups using Student’s t-test. Data are presented as mean ± SEM.

## Results

### Experimental Animals

Forty seven Romney ewe lambs commenced the study, 24 in the C Group and 23 in the U Group. Two lambs in the U Group became unwell with suspected parasitic infestation (weight loss, pallor suggestive of anemia) and were withdrawn from the study during the dietary intervention.

### Weight and Food Intake

Pre- and post-intervention weights were not different between groups (pre intervention C 30.3±0.7 kg, U 30.1±0.5 kg, p = 0.8; post intervention C 35.6±0.9, U 34.8±0.7 kg, p = 0.5). There was no difference between groups in total weight gain (expressed as % body weight at the commencement of the study) over the 6 week period (C 18±2%; U 16±2%; p = 0.4), which was within the range specified in the study design. Total food intake (amount supplied minus amount left over for the group, calculated daily) over 6 weeks was approximately 3% less in the Unpredictable Group (C 99.0±0.1 kg, U 95.0±0.5 kg per lamb; p<0.01).

### Glucose Tolerance

**Figure 1:**
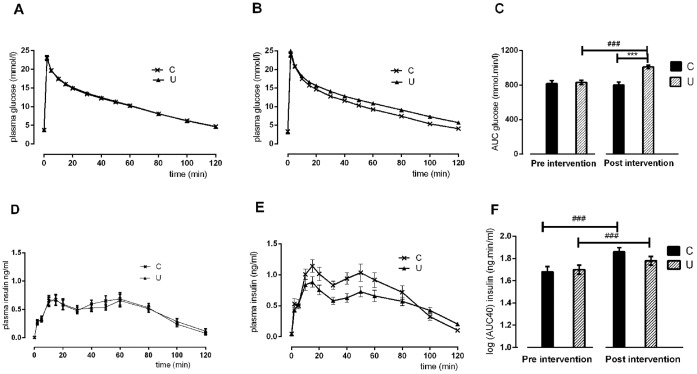
Glucose and insulin responses to intravenous glucose tolerance test. Plasma glucose (A,B) and insulin (D,E) concentrations and area under the curve (AUC) (C,F) during intravenous glucose tolerance test (IVGTT) before (A,D) and after (B,E) the six week dietary intervention, in regularly fed (C) and unpredictably fed (U) sheep. Values are mean±SEM. ***p<0.001 for difference between C and U groups ###p<0.001 for difference pre and post intervention within an experimental group Log(AUC40) Insulin = log area under the curve for insulin response in the first 40 minutes after the glucose bolus.

Before the experiment, glucose tolerance was similar in both groups (glucose AUC during IVGTT: C 818±34 vs U 830±25 mmol.min.l^−1^ (Figure 1 A,C): C insulin AUC 55±7 ng.min.ml^−1^, [log(insulin AUC) 1.68±0.05 ng.min.ml^−1^] vs U insulin AUC 55±5 ng.min.ml^−1^ [log(insulin AUC) 1.70±0.04 ng.min.ml^−1^]) (Figure 1 D,F). After the intervention, glucose AUC did not change in the C Group (801±33 mmol.min.l^−1^), but increased by 20% in the U Group (1010±19 mmol.min.l^−1^; p<0.0001 for comparison with C post-intervention and with U pre-intervention) (Figure 1B,C). Insulin AUC increased in both groups, but the increase was greater in the C group: C insulin AUC 79±9 ng.min.ml^−1^ [log(insulin AUC) 1.86±0.05 ng.min.ml^−1^; p<0.0001] vs pre-intervention; U insulin AUC 64±6 ng.min.ml^−1^ [log(insulin AUC) 1.78±0.04 ng.min.ml^−1^], p = 0.02 vs pre-intervention (Figure 1E,F). Compared with those in the C Group, post-intervention insulin concentrations in the U Group increased less after the glucose bolus to a lower first phase insulin peak (C insulin AUC first 40 minutes 32±3 ng.min.ml^−1^ [log(AUC insulin first 40 minutes) 1.49±0.04 ng.min.ml-1] vs U insulin AUC first 40 minutes 25±2 ng.min.ml^−1^ [log(AUC insulin first 40 minutes) 1.36±0.04 ng.min.ml^−1^], p = 0.03, so that glucose concentration in the U group failed to return to baseline at 120 minutes ([Baseline glucose concentration –120 min glucose concentration]: C 0.6±0.3 mmol.l^−1^, U 2.8±0.2 mmol.l^−1^, p<0.0001) (Figure 1E).

### Insulin Tolerance

**Figure 2:**
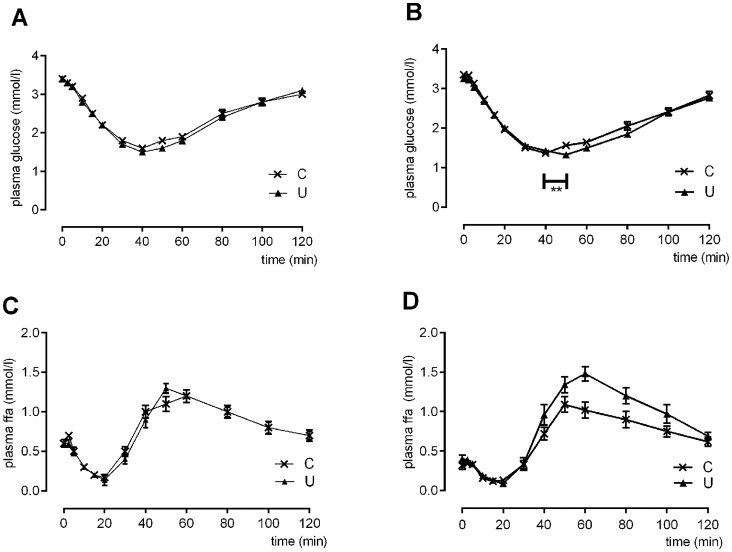
Glucose and free fatty acid response to insulin tolerance test. Plasma glucose (A,B) and free fatty acid (ffa)(C,D) concentrations during insulin tolerance test (ITT) before (A,C) and after (B,D) the six week dietary intervention in regularly fed (C) and unpredictably fed (U) sheep. Values are mean±SEM. ** p<0.01 for difference between C and U groups in the timing of nadir plasma glucose concentration. Time effect p<0.001, group × time interaction p<0.001; group effect p = 0.06 for plasma free fatty acid (ffa) concentrations after the intervention (D).

There was no difference in baseline glucose concentrations between groups before or after the intervention, and no change in baseline glucose concentrations in either group over time (Figure 2A,B). Nadir glucose concentrations also were similar in both groups before the intervention (Figure 2A). However, after the intervention, the time of the nadir was approximately 9 minutes later in the U Group than the C group (C 40±2 vs U 49±2 min, C vs U p = 0.003, U pre vs post-intervention p = 0.05) **(** Figure 2B **)**. The rate of decrease of glucose concentration after insulin injection was not different between groups before or after the intervention. However, in the C but not U group, the decrease was faster after the intervention than before (before, C slope -0.057±0.002 vs U -0.058±0.002 mmol.l^−1^.min, p = 0.5; after, C -0.065±0.002 mmol.l^−1^.min, p = 0.02 vs C pre-intervention; U -0.059±0.002 mmol.l^−1^.min, p = 0.7 vs pre-intervention; C vs U p = 0.09) (Figure 2A,B).

Free fatty acid (FFA) response to insulin was not different between groups before the intervention (Figure 2C). After the intervention, the U group had an earlier rise in plasma FFA concentration, and a greater, more sustained peak (group effect p = 0.06, time effect p<0.0001, group × time p = 0.0007) (Figure 2D).

### Diurnal Cortisol Concentrations

**Figure 3:**
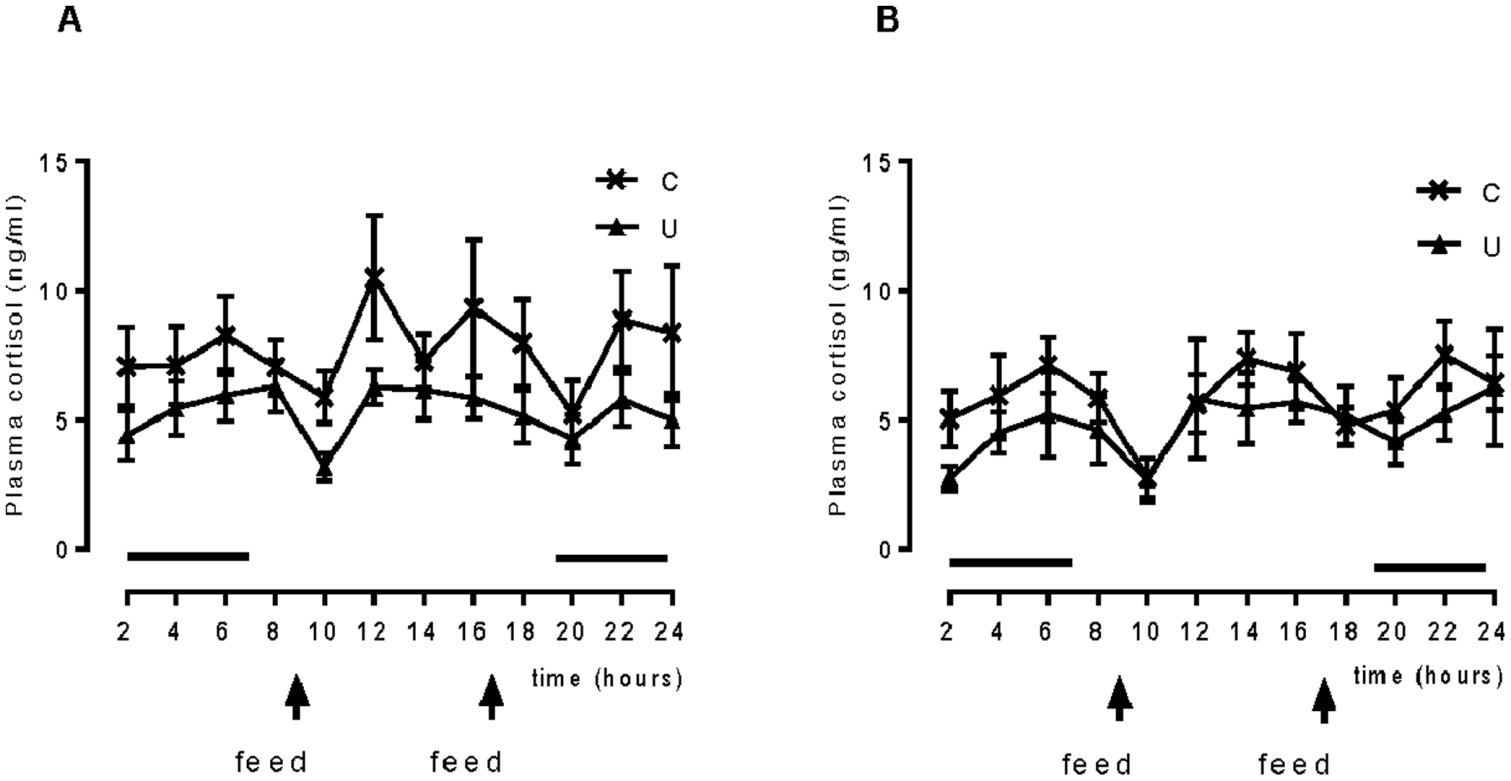
Effect of unpredictable feeding on diurnal cortisol concentrations. Diurnal plasma cortisol concentrations, before (A) and after (B) the six week dietary intervention in regularly fed (C) and unpredictably fed (U) sheep. Values are mean±SEM. There are no significant differences between groups. Solid lines parallel to the x axis reflect periods of darkness.

Plasma cortisol concentrations tended to be lower overall in the U Group both pre and post intervention (RM ANOVA group effect p = 0.05 for both time periods) (Figure 3). Cortisol variability was not different between groups (mean±SEM of SD pre-intervention: C 4.6±0.7, U 3.3±0.2, p NS; post intervention: C 3.5±0.3, U 3.1±0.6, p NS).

## Discussion

We have demonstrated that an unpredictable food supply results in impaired glucose tolerance in rapidly growing juvenile lambs, even when food quality is high and weight gain not excessive. This did not appear to be because of insulin resistance or increased circulating concentrations of stress hormones, but because of impaired insulin secretion. Although insulin response to a glucose load increased with age and growth in both groups, the increase was less in the unpredictably fed animals and inadequate to maintain normal glucose tolerance.

We hypothesized that making the food supply unpredictable would alter circadian rhythms and disrupt the co-ordinated metabolic response to a feed. Sheep were used preferentially over other animals such as rodents for several reasons: they are a large, primarily diurnal animal; there are well documented data regarding glucose metabolism and HPA axis function in sheep under a range of conditions; diurnal variation in hormones and metabolites occurs in response to changes in feed frequency, and it is possible to take serial blood samples. Many studies of the relationships between disrupted circadian rhythms and disordered metabolism in humans have focused on the sleep–wake cycle, rather than meals *per se* [28], [29]. A recent study investigating the metabolic effects of sleep deprivation and circadian disruption in adult human volunteers found relative hyperglycemia after the breakfast meal in sleep deprived subjects because of inadequate glucose-triggered insulin secretion, hypothesized to be due to desynchronization between the central circadian pacemaker and the response of circadian oscillators in peripheral tissues to sleep-wake and fasting-feeding cycles [28]. It is, therefore, interesting that a decreased insulin response to a glucose load also was found in the lambs in our study after the sole intervention of changing the feed regimen in an unpredictable way, without alteration in light-dark periodicity and with all feed episodes occurring within a 13 hour ‘daytime’ period.

In rodents, changes in feed pattern have been shown to affect both peripheral and central components of the circadian clock [30], [31]. Restricting the duration of food availability resulted only in disruption of circadian oscillators in peripheral tissue, rather than affecting the suprachiasmatic nuclei (SCN) in the hypothalamus, [17] while other nutritional and light-dark interventions have shown phase shifts in the central expression of certain clock genes [32].

Potential mechanisms underlying the decreased insulin response in our study could be via changes in the normal control of insulin release from the beta cell; or, alternatively, altered central regulation and hypothalamic-pancreatic signaling. Insulin secretion from the pancreatic β cell after a glucose load is pulsatile, reflecting oscillating metabolism within β cells, [33] and is dependent on glucose transport into the β cell, release of pre-formed insulin (first phase), and insulin synthesis and secretion (second phase) [34]. Given that both first and second phase insulin secretion were affected, possible changes in the pancreas include altered regulation of the β cell glucose sensor, glucokinase, or the glucose transporter responsible for the entry of glucose into the β cell, SLC2A2. Another possibility is the loss of normal pulsatility of insulin secretion, which occurs in type 2 diabetes, although the underlying mechanisms controlling this are poorly understood [35]. In sheep, as in other species, the metabolic response to food is also influenced by a cephalic phase induced by food-related sensory stimuli, mediating early release of insulin through hypothalamic signals via efferent vagal fibers to the pancreatic β cell [36]. Although giving a glucose load intravenously will not result in the same sensory cues as the presence of food, different nutritional states in sheep such as chronic undernutrition or high protein feeding alter secretion of hypothalamic hormones involved in appetite regulation [37]. The attenuated insulin response seen after the period of unpredictable feeding in our experiment could, therefore, reflect disruption of central influences on metabolism, or in-coordination between central and peripheral signals.

Key mediators of the physiological response to meals such as the orexigenic hormone ghrelin, released from the gut and acting centrally to enhance the early insulin response, may also contribute to altered glucose tolerance [34]. A transient ghrelin surge occurs just before a scheduled feed in sheep, [35] suggesting that secretion occurs in response to a cephalic mechanism, rather than because of direct contact with food. We were unable to measure ghrelin concentrations, but findings of a delayed insulin response to glucose and increased lipolysis after insulin in the U group may reflect either decreased ghrelin release or an attenuation of the response to ghrelin.

There is evidence that nutrition *in utero* modifies the development of appetite regulating pathways in the fetal hypothalamus, [38] but the effect of dietary intake and eating patterns on appetite control beyond the neonatal period is less clear. We were only able to study short-term outcomes, but it is conceivable that a mismatch between hunger and satiety, particularly if this occurs in an unpredictable pattern, may disturb entrainment of hypothalamic appetite regulatory pathways in young animals. In adults with type 2 diabetes, hypothalamic dysfunction has been demonstrated by functional MRI scanning, with failure of inhibition of neuronal activity by ingestion of a glucose load compared with healthy controls, although the identity of the neurones has not been established [39]. The authors concluded that failure of hypothalamic pathways to respond appropriately to increased glucose could impair post-prandial insulin action, and also affect satiety. If an unpredictable eating pattern in early life altered the co-ordination of food intake, hypothalamic response, and perception of hunger, this might be a contributing mechanism to the later development of metabolic disease, including obesity.

A stress-related rise in plasma cortisol concentration after feed delay has been previously demonstrated in sheep, [40] and studies in adult humans have shown an association between irregular eating and insulin resistance. We therefore hypothesized that disruption of diurnal cortisol secretion would underlie any metabolic effects of unpredictable eating, resulting in cortisol-induced hyperglycemia and insulin resistance. However, this hypothesis was shown to be incorrect, as our animals showed neither increased cortisol secretion nor insulin resistance. Both these negative findings are important and may indicate a difference in the response to irregular eating in young, healthy growing animals compared with adults [4]. In our study, we used an insulin tolerance test as an indicator of insulin sensitivity for logistic reasons, rather than the more definitive hyperinsulinemic-euglycemic clamp. However, the lack of difference between groups in the fall in glucose concentration after an insulin bolus, together with decreased insulin secretion after a glucose load in the U group indicate that insulin resistance is unlikely to have contributed to the glucose intolerance seen in our animals.

Sheep are ruminants with grazing as the natural feed pattern. Blood glucose concentrations are maintained by the ongoing metabolism of volatile fatty acids (VFAs) released by the action of microflora in the rumen [41]. One would, therefore, expect that sheep would be physiologically more stable in the face of an irregular food supply than humans or rodents, as the prolonged fermentation of large amounts of cellulose and hemicellulose in the rumen would continue to produce VFAs during brief periods of food absence, possibly masking the effects of irregular feeding, and a potential limitation to the study. Despite this, we found that even a relatively short period of unpredictable feed supply resulted in clear metabolic effects. The animals were housed in groups, rather than individual pens, with the obvious limitation that feed intake of an individual animal was not able to be measured. However, feed intake in all animals was clearly adequate for growth. The positive aspects of housing the animals in group pens were more normal interactions between animals and more spontaneous activity than would have been possible in individual pens for such a prolonged period. The main aim of this experiment was to investigate the metabolic effects of an unpredictable eating pattern; these outcomes are unlikely to have been affected by group feeding *per se*. However, the proximity of the group pens meant that groups were able to see each other, with the possibility of anticipatory hormonal changes in both groups when seeing the other group being fed [42]. This would have been expected to dilute, rather than augment, the magnitude of any differences caused by unpredictable feeding. A further limitation was that metabolic responses to altered feeding may be different between sexes, and for logistic reasons we were only able to study females.

### Conclusions

Our findings suggest that an unpredictable food supply in young growing animals affects metabolism in ways that impair glucose tolerance, even when food is of good quality and in the absence of excessive weight gain. These effects may be different in the young from those in adults, and are likely to be mediated via central mechanisms that co-ordinate food intake, satiety and post-prandial metabolism, although peripheral oscillators in the pancreas may also be affected. Reversibility has yet to be established. Unpredictable eating patterns that vary from day to day, particularly in young children, warrant consideration in the human population as a potentially remediable contributor to disordered glucose metabolism.
